# Targeted Metabolic Profiling Indicates Apple Rootstock Genotype-Specific Differences in Primary and Secondary Metabolite Production and Validate Quantitative Contribution From Vegetative Growth

**DOI:** 10.3389/fpls.2018.01336

**Published:** 2018-09-21

**Authors:** Rachel Leisso, Dave Rudell, Mark Mazzola

**Affiliations:** ^1^Montana State University Western Agriculture Research Center, Corvallis, MT, United States; ^2^Physiology and Pathology of Tree Fruits Research, Agricultural Research Service (ARS), United States Department of Agriculture (USDA), Wenatchee, WA, United States

**Keywords:** rhizodeposits, root exudates, *Malus domestica*, rootstocks, G935, M26, metabolomics

## Abstract

Previous reports regarding rhizodeposits from apple roots are limited, and complicated by microbes, which readily colonize root systems and contribute to modify rhizodeposit metabolite composition. This study delineates methods for collection of apple rhizodeposits under axenic conditions, indicates rootstock genotype-specific differences and validates the contributions of vegetative activity to rhizodeposit quantity. Primary and phenolic rhizodeposit metabolites collected from two apple rootstock genotypes, G935 and M26, were delineated 2 months after root initiation by utilizing gas chromatography/liquid chromatography—mass spectrometry (GC/LC-MS), respectively. Twenty-one identified phenolic compounds and 29 sugars, organic acids, and amino acids, as well as compounds tentatively identified as triterpenoids were present in the rhizodeposits. When adjusted for whole plant mass, hexose, erythrose, galactose, phloridzin, kaempferol-3-glucoside, as well as glycerol, and glyceric acid differed between the genotypes. Phloridzin, phloretin, epicatechin, 4-hydroxybenzoic acid, and chlorogenic acid were among the phenolic compounds found in higher relative concentration in rhizodeposits, as assessed by LC-MS. Among primary metabolites assessed by GC-MS, amino acids, organic acids, and sugar alcohols found in relatively higher concentration in the rhizodeposits included L-asparagine, L-cysteine, malic acid, succinic acid, and sorbitol. In addition, putative ursane triterprenoids, identified based on accurate mass comparison to previously reported triterpenoids from apple peel, were present in rhizodeposits in high abundance relative to phenolic compounds assessed via the same extraction/instrumental method. Validation of metabolite production to tree vegetative activity was conducted using a separate set of micropropagated trees (genotype MM106) which were treated with a toxic volatile compound (butyrolactone) to inhibit activity/kill leaves and vegetative growth. This treatment resulted in a reduction of total collected rhizodeposits relative to an untreated control, indicating active vegetative growth contributes to rhizodeposit metabolites. Culture-based assays indicated an absence of bacterial or fungal endophytes in roots of micropropagated G935 and M26 plants. However, the use of fungi-specific primers in qPCR indicated the presence of fungal DNA in 30% of the samples, thus the contribution of endophytes to rhizodeposits cannot be fully eliminated. This study provides fundamental information for continued research and application of rhizosphere ecology driven by apple rootstock genotype specific rhizodeposition.

## Introduction

Rhizodeposits, which is the term used to encompass all plant metabolites entering the rhizosphere and originating from roots, are an important determinant of the rhizosphere microbiome composition and function which contributes to plant protection from pathogens and nutrient uptake (Berendsen et al., 2012) as well as plant growth (Vacheron et al., 2013). Furthermore, rhizodeposits alone have important impacts in chemical communication among plants and below-ground insect herbivores (Brink, 2016), can alter soil pH (Hinsinger et al., 2003), and nutrient availability (Shahbaz et al., 2006).

Previous work demonstrated that rhizodeposits can differ among apple rootstock genotypes (Leisso et al., 2017). However, in previous experimentation, despite extensive disinfestation of plant material and growth media, microbes rapidly colonized tree roots and were readily cultured from water-percolated rhizodeposits. The numbers of culturable microbes recovered from individual potted plant systems correlated well with tree leaf area and were significantly greater than no-tree pot controls. The cultured microbes were apparently largely benign, as vegetative growth appeared normal; no wilting or discoloration of leaves was observed. Leaf size and leaf area as well as the quantity of cultured microbes corresponded with currently understood concepts of relative genotypic vigor among the four tested rootstock genotypes (M26 > G935 > G41 > M9Nic29, in terms of tree size). Collected rhizodeposits differed according to rootstock genotype (Leisso et al., 2017). Analysis of these rhizodeposits suggested the presence of metabolites of non-tree origin, based on accurate mass search in the METLIN library (Smith et al., 2005), thus a small set of trees were axenically propagated to enable validation of tree-originating metabolites, as reported in the study. The present study expands the number of axenically propagated trees assessed and the number of metabolites evaluated, and further reports the relative difference in metabolite quantity between the rhizodeposits of two genotypes in the absence of microorganisms that could influence metabolite composition.

The importance of differentiating apple rhizodeposits stems from research indicating genotype-specific apple rhizosphere microbiomes (Mazzola and Manici, 2012; Reed and Mazzola, 2015), whose communities can have functional differences in terms of disease suppression and plant growth benefits. From the plant side, genotype specific genes (Fazio et al., 2011) and gene expression (Zhu et al., 2014) have been separated, especially in terms of root disease tolerance and response to necrotrophic plant pathogens (Zhu et al., 2016). In apple rootstocks' roots, Shin et al. (2016) detected changes in gene expression related to biosynthesis of phenolic compounds including flavanols, chalcones (e.g., phloridzin), and leucoanthocyanindins. Gene expression related to phenolic production has also been demonstrated to differ among heterogenous genotypes in a time-dependent fashion among varieties of the same genus (Sharma et al., 2016). Applied management topics linked to understanding rhizosphere microbiomes include promoting a microbiome providing tolerance to apple replant disease (ARD) (Mazzola and Freilich, 2016), tree nutrition, and the potential for site-driven rootstock genotype selection.

The objective of this study was to determine the influence of rootstock genotype on qualitative and quantitative attributes of apple rhizodeposits. Rhizodeposits from axenically reared apple rootstocks were assessed for phenolic compounds, organic acids, amino acids, sugar alcohols, sugars, and triterpenoids and the relative differences between the two apple rootstock genotypes was determined.

## Methods

### Experimentation

The first experiment assessed overall method viability and compared rhizodeposit metabolic profiles of 5 replicates each of M26 and G935; only phenolic compounds were assessed in this experiment. One replicate was removed from each genotype from the first experiment, due to microbial contamination. In the second experiment, experimentation was set up for 9 replicates for each of M26 and G935; two replicates were removed from each genotype due to microbial contamination in two replicates of the M26 rhizodeposit collection. Phenolic compounds, certain triterpenoids, as well as sugars, amino acids, organic acids, and sugar alcohols were assessed in this second experiment. A validation experiment (experiment three), assessed root tissue from micropropagated trees for endophytes with 5 separate G935 and M26 plantlets from the same initiation lines, due to the need to retain whole plantlets from second experiment for metabolic dry weight correction. A further validation experiment (experiment four) to ascertain the contribution of live plant metabolic activity to rhizodeposits (as opposed to sloughed off callus and root cells) was performed with twelve MM106 plantlets due to limited availability of G935 and M26. Micropropagation and rhizodeposit collection were similar for all experiments as described in the following sections.

### Micropropagation

Micropropagated rootstock trees (M26, G935, and MM106) were grown axenically with methods similar to Dobranszki and da Silva (2010); Sun et al. (2016) and Yepes and Adwinckle (1994). Shoots were grown in sterile Magenta boxes (GA-7, Sigma Aldrich) in a growth chamber (12 h light at 25°C/ 12 h dark 20°C) in shoot multiplication media (SMM) to the height of ~4 cm, and then sterilely excised from the callus and transferred to rooting induction media (RIM). Previous assessment of light source indicated that under the experimental conditions employed, photosynthetically active radiation was supplied at 215 μmol s^−1^ m^−2^. Shoots were maintained in rooting media for 1 week in the dark prior to transferring to root elongation media (REM). Once transferred to REM, roots proceeded to emerge and were allowed to extend until encircling the base of the Magenta box (2 months ± 1 week).

### Rhizodeposit collection

**Figure 1:**
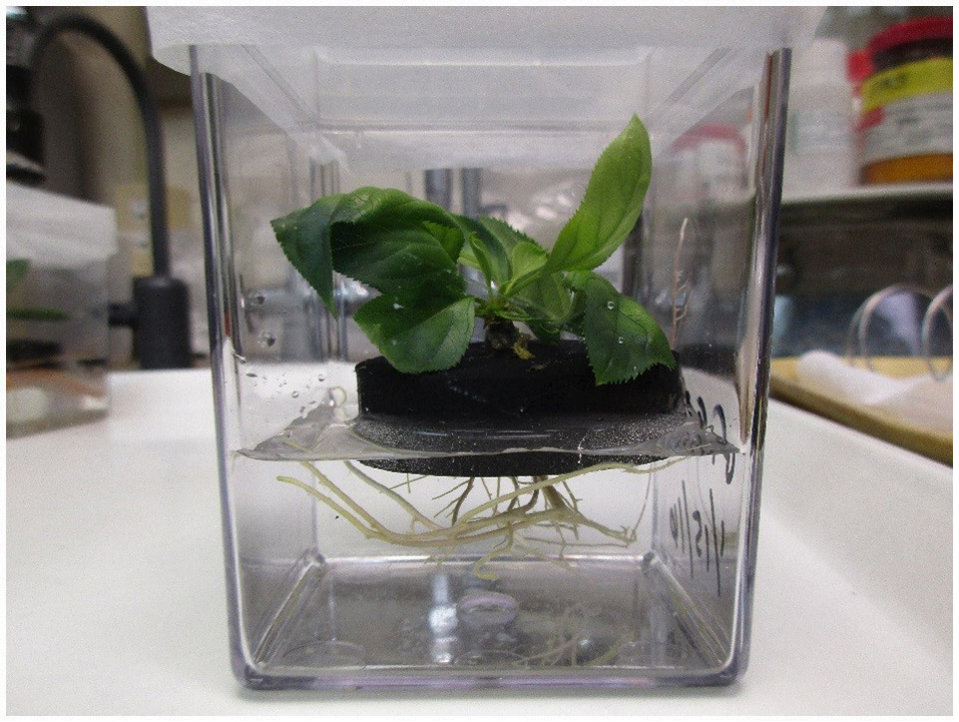
Example experimental unit: rooted axenically micropropagated rootstock on sterilized neoprene float. Trees remained in water for 5 days after which water was filtered, flash frozen, and subsequently processed for metabolite analysis.

Trees were removed from root elongation media, and roots were rinsed in sterile distilled water to remove loose callus tissue. The tree was then placed in a 2″ circular neoprene float (Ehydroponics.com) which had been disinfested by soaking in 95% ethanol for 24 h, and dried on a sterile Petri plate in a laminar flow hood. The tree was then transferred to a sterile Magenta box containing 85 mL sterile distilled water to allow remaining callus tissue to slough off. After 24 h, the tree was transferred again to a new Magenta box containing 85 mL sterile distilled water, where it remained for 4 additional days (Figure 1) at which time a 100 μl aliquot was plated to one-tenth strength tryptic soy agar (TSA) to assess bacterial contamination. The rhizodeposit sample was filtered through glass wool packed in a 60 mL syringe and frozen to −80°C immediately. After 3 days at 25°C, any samples that exhibited bacterial growth on TSA plates were removed from the freezer and discarded.

### Rhizodeposit sample processing

Rhizodeposit samples were processed using previously described methods (Leisso et al., 2017) with an added step for organic acid, amino acid, and sugar analysis sample processing. Rhizodeposits were frozen and stored at −80°C, and lyophilized (Unitrap II, Virtis, Gardiner, NY) until water removal was complete (5 days). After lyophilization, rhizodeposits were resuspended in 3 ml methanol. Samples were vortexed for 30 s, and 500 μl was removed for GC-MS analysis (derivatization described below). The remaining rhizodeposits were then dried under a stream of nitrogen gas and resuspended in 200 μl of methanol and filtered (PVDF membrane, 0.22 um pore size, 4 mm diameter, Millex, EMD Millipore, Billerica, MA) immediately prior to sample injection for LC-MS QTOF analysis. Control samples of 1 cm^2^ root elongation media (REM) dissolved in 85 mL water as well as neoprene floats alone in water were processed in the same manner in order to enable subtraction or exclusion of any metabolites also originating from these sources.

### LC-MS QTOF analysis

For the first and fourth experiment, samples were analyzed for phenolic compounds using a method previously described in Leisso et al. (2017). The second experiment used an extended run length and different HPLC column to enable analysis of more metabolites, including several tentative triterpenoid compounds. The list of targeted metabolites confirmed by authentic standards and analyzed by each LC-MS method are indicated in Supplemental Materials (Supplemental Table 1 C18 short targeted compounds.xlsx; Supplemental Table 2 C18 long targeted compounds.xlsx). For the longer method, concentrated rhizodeposit samples were analyzed for metabolite composition using an Agilent 1260 HPLC equipped with a 6520 Accurate-Mass Q-TOF LC/MS (Agilent Technologies, Inc., Santa Clara, CA). Ten microliter of sample was injected. Solvent flow rate was 1 min mL^−1^ through an endcapped C18 reverse phased column (Chromolith Performance RP-18e), with a 4.6 mm internal diameter and 100 mm length (EMD Millipore, Merck, KGaA, Darmstadt, Germany). The solvent temperature during resolution was maintained at 25°C. The solvent system was based on previously published methods for phenolic analysis in apple (Schieber et al., 2001). Solvent A consisted of 2% acetic acid (Fisher Scientific, Fairhaven, NJ) in HPLC grade filtered water. Solvent B consisted of 0.5% acetic acid in 1:1 (v/v) acetonitrile (HPLC grade, Sigma-Aldrich, St. Louis, MO) and HPLC grade filtered water. The solvent system was 0–50 min, 90–45% A gradient; 50–70 min, 45–0% A gradient; 70–80 min, 0–90% A gradient, and 12 min 90% A for column re-equilibration. The ion source used was ESI in negative ion polarity mode, and only MS data between mass ranges between 70 and 1,200 m/z were recorded, at an acquisition rate of 1 spectrum/s and 1,000 ms/spectrum. The drying gas temperature was 350°C at a flow rate of 4 min L^−1^. The nebulizer gas pressure was 60 psig. The voltage cap was 3,000 V, the fragmentor, 125 V, the skimmer, 65 V, and the octopole, 750 V. The instrument was calibrated before each run with ESI-Low Concentration Tuning Mix (Agilent Technologies, Inc.), and during each run the reference masses 119.0360 and 980.0163 (G 1969-85001 ES-TOF Reference Mass Solution Kit, Agilent Technologies Inc.) were used, prepared per protocol in Agilent 6500 Q-TOF LC/MS maintenance guide. The auto recalibration reference mass parameters were 100 ppm with a minimum of 1,000 counts.

Additionally, samples in the fourth experiment (*Contribution of live plant metabolic activity to rhizodeposits*, detailed below) were also analyzed with an LC-MS method using a zicpHILIC column for quantifying organic acids, amino acids, sugars, and other polar compounds as described in Rudell et al. (2017).

### Derivatization and GC-MS analysis

Methoxyanimation/trimethysilylation for analysis of sugars, organic acids, amino acids, and sugar alcohols was performed essentially as described by Rudell et al. (2008). Derivatized extract (0.2 μL) was injected into a 6890N GC coupled with a 5975B mass selective detector (MSD) using a 7683B automatic injector (Agilent Technologies, Palo Alto, CA). Samples were volatized in a 230°C splitless inlet lined with an unpacked 4 mm internal diameter, deactivated, tapered-bottom glass liner. Further focusing of the sample was accomplished using a pulsed injection technique that maintained a He carrier gas linear velocity of 66 cm s ^−1^ for the first 0.25 min, reducing it to 40 cm s^−1^ thereafter. The GC column was a HP-5MS (Agilent Technologies) (30 m × 250 μm × 0.25 μm). The oven initial temperature was 40°C held for 2 min followed by 18°C min^−1^ increase to a final temperature of 330°C that was held for 6 min. The detector was operated in EI mode with transfer line, source, and quadrupole temperature maintained at 250, 150, and 230°C, respectively. Mass spectra ranging from m/z 30 to 500 were recorded.

Samples were analyzed a second time with a 20:1 split injection in order to better resolve the major sugars.

### MZmine analysis of LC-MS QTOF data

Agilent data files were converted to mzdata files from MassHunter software. Mzdata files were analyzed in MZmine (v 2.27) (Pluskal et al., 2010). Raw data files were analyzed utilizing either a targeted peak detection procedure based on a library created from previously run authentic standards, an untargeted library generated from the data set, or both. Targeted peak detection was used for experiment one and two to enable assessment of specific compounds; untargeted analysis was the primary method performed for experiment four, as it was a validation experiment simply assessing the totality of collected rhizodeposits, and would represent a greater number of (unidentified) compounds than targeted methods. Targeted peak detection libraries were created using retention times and accurate masses of authentic standards or compounds whose identity was partially characterized on the basis of accurate mass/spectral information in conjunction with literature review of apple compounds (Supplemental Table 1 C18 short targeted peak list.xlsx and Supplemental Table 2 C18 long targeted compound list.xlsx). Settings for all analyses are further detailed in Supplemental Materials (Supplemental Table 3 MZmine settings Micropropagation.xslx).

### Chemstation/deconvolution reporting software analysis of GC-MS TMS oxime data

Derivitized sample datafiles from GC-MS analysis were processed in Chemstation software (Agilent Inc., Palo Alto, CA, USA). Separate analysis methods were applied to split vs. splitless injections, with split injections focusing on peaks which did not have good separation in the splitless injection due to high abundance, specifically malic acid, fructose, glucose, sorbitol, and myo-inositol. The library from the splitless injection included amino acids, ursane triterpenoids, organic acids, sugar alcohols, and other sugar species; libraries employed were previously reported in Leisso et al. (2013). Some metabolites were identified with co-elution to authentic standards, while others were identified with mass spectral tags (mass and retention indices). Peak areas in Chemstation were obtained utilizing the deconvolution reporting service (DRS) which uses the algorithm from AMDIS software (Agilent Inc.,) which determines peak area specifically for the ions in the mass spectra. Compound peak area was then adjusted for total dry plant weight before statistical analyses.

Fructose, glucose, myo-inositol, and L-alanine were found in high concentrations in extraction of fresh REM media (in which plantlets had been grown; the concern was that small amounts could remain on the roots, despite rinsing procedures) and were excluded from further analyses.

### Assessment of micropropagated trees for endophytes

Roots of micropropagated G935 and M26 cultivars were assessed to determine the presence of endophytes in the axenically micropropagated trees which could influence metabolic profiling results. As trees were initiated in micropropagation media from actively growing foliar shoot tips, endophytes present in the roots would have had to originate from foliar tissue.

Trees were in root elongation media longer than the usual rooting window (3 months) in order to further allow any endophyte or contaminant growth.

Tissue collection was performed in the laminar hood. Trees were extracted from root elongation media, and any remaining media was removed from the roots with flamed-sterilized forceps. Roots and shoots of trees (5 per genotype M26 and G935) were axenically divided and placed in sterile specimen cups and flash frozen by placing the closed container in liquid nitrogen. Root tissue was chopped finely and DNA was extracted from a 50 mg sample using the PowerPlant Pro DNA isolation kit (MoBio Laboratories Inc.,) and a final elution volume of 50 μl. DNA was extracted from roots of a micropropagated tree exhibiting fungal contamination and used as a positive control in PCR reactions detailed below.

Total fungal and bacterial populations in root tissue were evaluated using a real-time qPCR procedure using the primers and amplification conditions as previously described (Reardon et al., 2013). Both 10 and 100 time dilutions of the root DNA were used as template in the amplification reactions.

The presence of culturable fungi or bacteria in roots of asymptomatic (no hyphae or bacterial colonies obvious in the media) micropropagated trees of cultivar G935 and M26 was assessed using full strength PDA (24 g L^−1^ potato dextrose broth + 15 g L^−1^ agar) and full strength TSA (30 g L^−1^ tryptic soy broth + 15 g L^−1^ agar), respectively. Five root segments from different roots were plated to TSA and PDA; the main stem was cut into 5 segments; each segment was streaked on TSA and then gently placed in the surface layer of the PDA media.

### Contribution of live plant metabolic activity to rhizodeposits (butyrolactone treatment)

**Figure 2:**
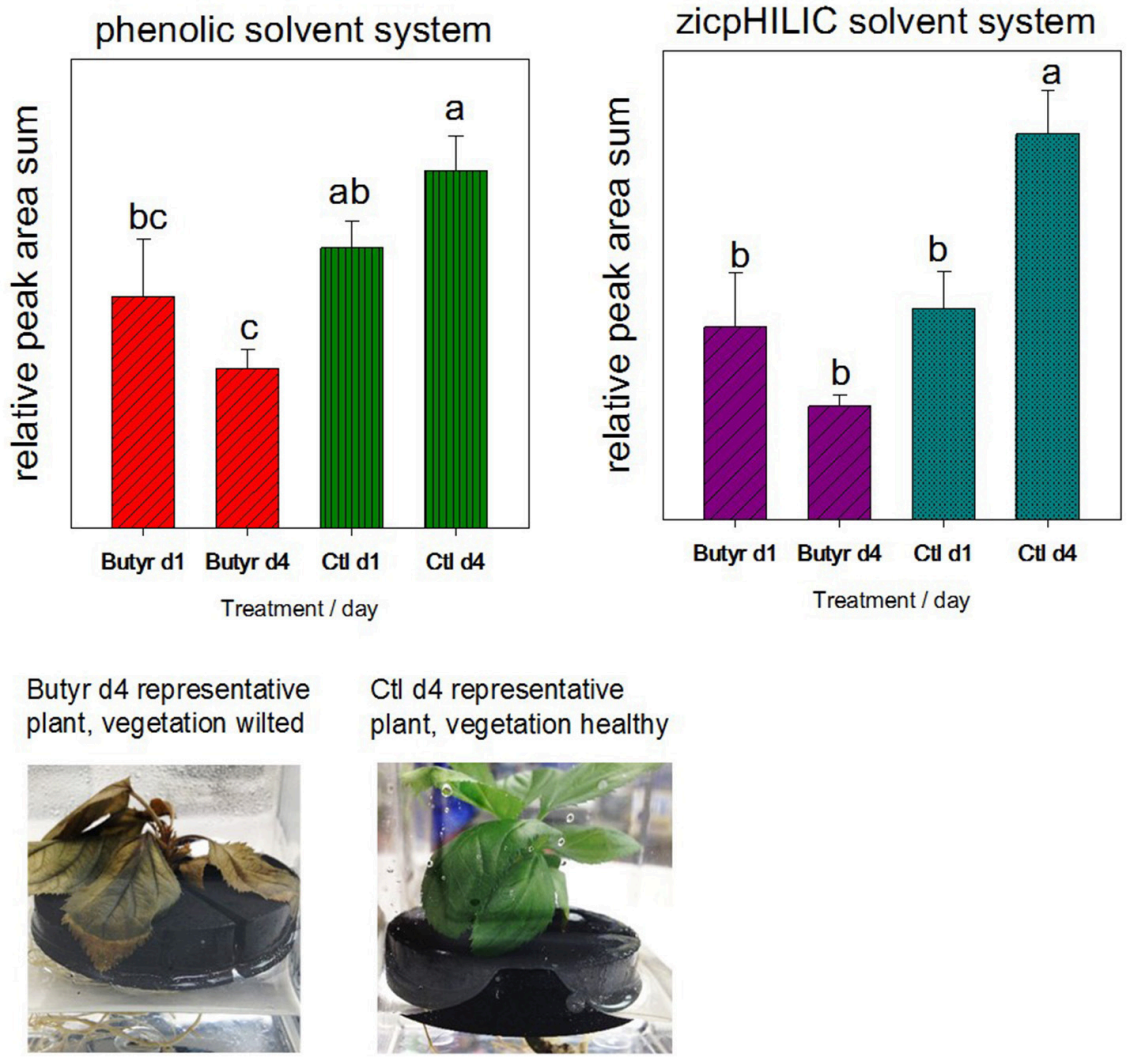
Validation of rhizodeposit metabolites collected via root dip originating in connection with vegetative growth. The concern was that sloughed off root or callous cells would contribute more greatly to rhizodeposits than other modes of rhizodeposition. After rinse and soaking steps, 12 micropropagated trees (genotype MM106) were divided into treatment groups (butyrolactone [butyr] and untreated control [ctl]), incubated for another day in water, then rhizodeposits collected (d1). Following rhizodeposit collection (d1) and transfer to new water, micropropagated trees were treated with the toxic volatile compound butyrolactone to kill/reduce growth of vegetation, and after 4 days in new water, rhizodeposits collected (d4). Differing letters (a–c) above bars indicate statistically significant means.

To assess the potential contribution of sloughed off cells to the rhizodeposit metabolome, a follow-up validation experiment was performed, where plants were treated with a toxic volatile compound to slow growth and destroy functionality of vegetative tissue. The study employed MM106 rootstock due to insufficient quantities of G935 or M26 plants. Twelve micropropagated explants (MM106) in water were rinsed and transferred from media to neoprene floats as describe above; after a day of soaking to remove callus tissue, explants were transferred to fresh sterile distilled water, and water collected for rhizodeposit analysis after an additional day. Following the initial collection, explants were transferred to fresh collection boxes, and for half (6) of the trees, a sterile filter disc treated with 10 μl butyrolactone (Sigma-Aldrich), a toxic volatile compound produced by *Phytophthora cinnamoni* (Qiu et al., 2014) was placed on top of the neoprene float beneath the leaves of the explants. Ninety-six hours after experiment initiation, trees treated with butyrolactone appeared wilted (Figure 2), and rhizodeposits were collected and frozen with an aliquot of rhizodeposit liquid plated to 50% TSA to check for contamination. Trees were placed whole in a drying oven for subsequent dry weight correction for rhizodeposit metabolites. Rhizodeposits were analyzed for both untargeted phenolic compounds per the LC-MS phenolic method as described above and other untargeted polar compounds using the zicpHILIC method detailed in Rudell et al. (2017).

### Data analysis

Peak areas were normalized using the ibuprofen internal standard as a reference feature, and data were scaled using autoscaling (mean-centered and divided by the standard deviation of each variable) in MetaboAnalyst 3.0 (Xia et al., 2012). Data were analyzed in MetaboAnalyst using principal components analysis (PCA) and clustering (Euclidean algorithm, Ward's distance measure).

*T*-tests and ANOVA were performed in SAS with general linear models. As there were only two time points, ANOVA was used for butyrolactone/live plant contribution to rhizodeposit validation as opposed to a time series analysis.

## Results

### Amino acids, organic acids, sugar alcohols, and other compounds

Sugars, organic acids, amino acids, and sugar alcohols were analyzed via GC-MS following derivatization of lyophilized samples (Table 1). Among five amino acids assessed in rhizodeposits, L-asparagine and L-cysteine were detected at the highest quantity and only L-aspartic acid differed significantly between the two rootstock genotypes (higher in rhizodeposits from G935). Organic acids found in relatively high concentration included malic acid, succinic acid, malonic acid, glyceric acid, and oxalic acid. Succinic and glyceric acid levels were significantly higher for G935 rhizodeposits, while malonic acid was higher in rhizodeposits from M26. The sugars hexose, galactose, and erythrose were higher in the rhizodeposits from G935. Levels of several sugar alcohols were assessed but no significant difference existed between the two rootstocks and sorbitol was detected at the highest quantity among those assessed.

**Table 1:** Metabolites assessed via derivatization and GC-MS analysis included amino acids, organic acids, sugars, sugar alcohols, and other compounds.

**Compound (parentheses indicate identification is not confirmed by authentic standards); trimethylsilylation [TMS] indicates derivatized form for GC/MS**	**Retention time (min)**	**Quantification ion (m/z)**	**G935 rhizodeposit peak area g^−1^ dry weight whole plant**	**M26 rhizodeposit peak area g^−1^ dry weight whole plant**	“^*^” Significance at *p* < 0.05 between genotypes
**AMINO ACIDS**					
L-asparagine [3TMS]	11.2	159	2891380	2764796	
L-cysteine [3TMS]	11.4	406	2157648	1412312	
L-aspartic acid [3TMS]	10.7	232	848542	196042	^*^
B-alanine [3TMS]	10.0	160	557381	175848	
L-valine [2TMS]	8.4	144	479378	366937	
**ORGANIC ACIDS**					
Malic acid [3TMS]	10.5	335	6127902	5718066	
Succinic acid [2TMS]	9.1	147	1179148	497640	^*^
Malonic acid [2TMS]	8.3	147	617007	1337261	^*^
Oxalic acid [3TMS]	7.8	235	325887	615729	
Galacturonic acid [1TMS]	15.6	375	217022	186028	
Lactic acid [2TMS]	6.9	117	210143	171185	
Glyceric acid [3TMS]	9.3	315.0	104137	71080	^*^
Pyruvic acid [1TMS]	7.0	695	92595	51891	
**SUGARS**					
Hexose [5TMS]	13.8	319	574733	214959	^*^
Gentiobiose [8TMS]	17.8	361	463491	1467215	
Xylose [4TMS]	11.7	307	386442	429090	
Gulose [5TMS]	16.0	217	382891	183284	
Ribose [4TMS]	11.8	307	381419	468254	
Rhamnose [4TMS]	12.1	217	377152	408940	
Galactose [5TMS]	15.2	319	116357	230342	^*^
Erythrose [5TMS]	10.2	317	108654	81221	^*^
Ribofuranose [4TMS]	16.8	509	13591	165	
**SUGAR ALCOHOLS**					
Sorbitol [6TMS]	13.4	319	41071653	31450752	
Erythritol [4TMS]	13.7	423	1228877	892587	
Threitol [1TMS]	10.9	307	737590	542915	
Galactitol [6TMS]	15.6	421	57379	47484	
**MISCELLANEOUS**					
(Urea^**^) [2TMS]	8.5	189	12951401	12407485	
Glycerol^**^ [3TMS]	8.8	307	6408323	2320712	^*^
Ursolic acid [2TMS]	22.4	42	1278650	2681073	
(Phosphoric acid^**^) [3TMS]	8.9	189	659084	1338218	^*^
(Ursane2of4) [3TMS]	21.4	320	142450	479239	
(Octadecanoic acid^**^) [1TMS]	14.8	341	78556	91641	
(Ursane3of4) [2TMS]	22.1	571	16241	40512	
(Oleonitrile^**^) [no TMS]	16.0	319	14467	20213	
(Ursane1of4) [2TMS]	21.0	482	3816	26582	

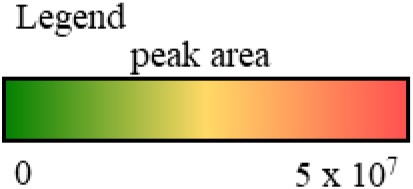

*Color coding in peak area cells indicates relative concentration*.

** Identity based on mass spectra and retention indices comparison to NIST library. Significance at p < 0.05 between genotypes (^*^).

**Figure 3:**
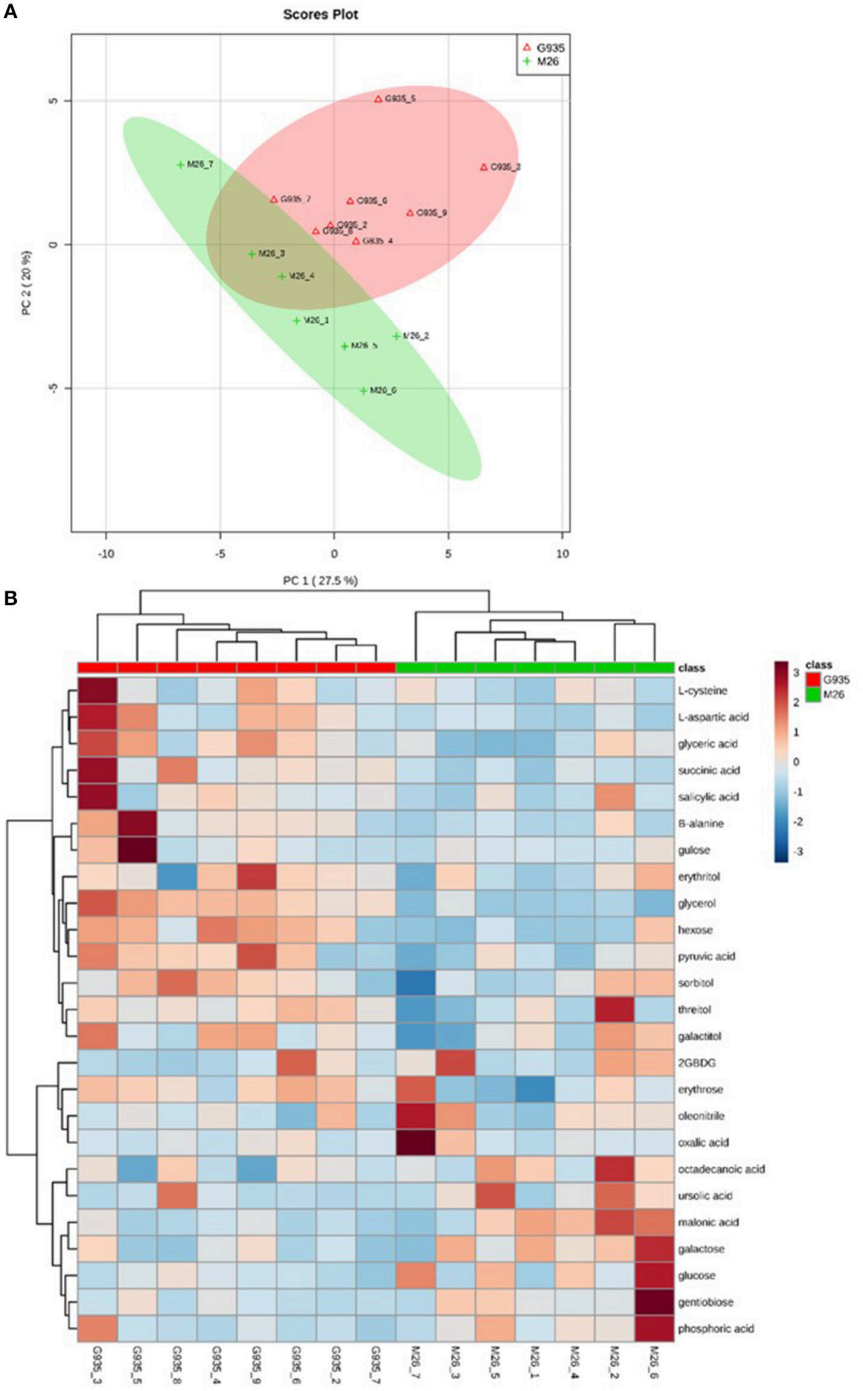
Metabolic analysis of compounds analyzed via metabolite derivitization (tri-methylsilylation) and GC-MS analysis, which includes organic acids, amino acids, sugars, and sugar alcohols. Principal components analysis **(A)** and clustering/heatmap visualization of top 25 compounds differing according to *t*-test **(B)** of relative compound levels indicates separation of the two cultivars assessed.

PCA/cluster analysis of combined metabolic profiles of sugars, organic acids, amino acids, and sugar alcohols indicated a general difference in relative quantity of many compounds assessed according to rootstock genotype (Figure 3A). Relative metabolite abundance among individual samples also illustrates that tree-to-tree rhizodeposit heterogeneity exists (Figure 3B). Greater heterogeneity among M26 samples is suggested by the broader distribution of samples in the PCA (Figure 3A).

Several metabolites detected in rootstock rhizodeposits compounds analyzed were also found in the agar-based media in which trees rooted prior to rhizodeposit collection, with the major metabolites including fructose, glucose, myo-inositol, and L-alanine. Every effort was made to remove media from the roots, including an overnight “soak” which had the dual purpose of callus tissue removal. However, although these metabolites could also be part of the apple rhizodeposit suite of metabolites based on reports of rhizodeposits from other plant species (Badri and Vivanco, 2009), in the present study they were excluded from further analysis.

### Phenolic and putative triterpenoid compounds

In the first experiment, only one phenolic compound (phloridzin) statistically differed in relative quantity between rootstocks; it was higher in M26 (data not shown).

**Figure 4:**
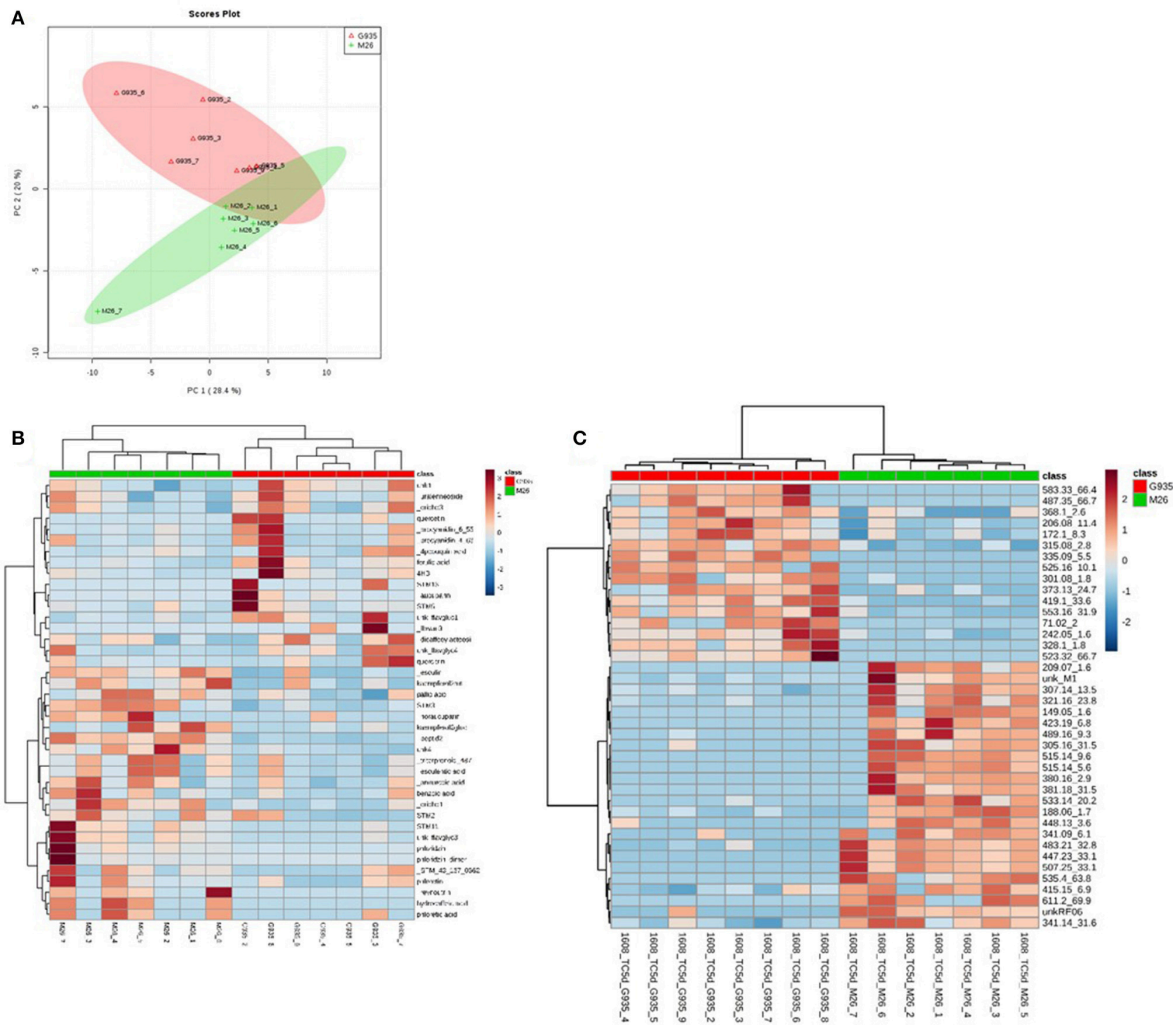
Principal components analysis **(A)** of rhizodeposit phenolic compounds and heatmap showing top 40 identified compounds according to *t*-test significance **(B)** and unidentified compounds **(C)**, also according to *t*-test significance, indicating separation of the cultivars according to genotype, as well as demonstrating the number of unidentified compounds differing between the genotypes in the LCMS method for phenolic compounds.

The identified compounds that differed significantly (*P* < 0.05) between G935 and M26 (normalized for plant dry weight and internal standard) in the second experiment included phloridzin, kaempferol-3-glucoside, two compounds with tentative identification based on mass spectral characteristics (a putative chlorogenic acid, esculin, and a 4-hydroxybenzoic acid fragment) (Table 2) as well as numerous compounds identified primarily by accurate mass and retention time (Figure 4). In collected rhizodeposits, putative triterpenoids, epicatechin, phloridzin (in M26), phloretin, and quinic acid had the highest relative levels, while rutin, phloroglucinol, and phloretic acid were detected at comparatively lower levels.

**Table 2:** Metabolites analyzed by LC-MS analysis included compounds tentatively identified as triterpenoids, flavonoids, chlorogenic acids, benzoic acids, procyanidins, and other phenolic compounds.

**Compound (paratheses indicates ID not confirmed by authentic standard)**	**Expected monoisotopic mass of pseudomolecular ion *m/z* [M-H]-, based on chemical formula**	**Experimental *m/z* [M-H]-**	**ppm monoisotopic mass difference (expected - experimental)**	**Retention time (min)**	**Abbreviation in heatmaps; “_” before name indicates identity not confirmed by authentic standard**	**G935 average peak area g^−1^ dry weight whole plantlet**	**M26 average peak area g^−1^ dry weight whole plantlet**	“^*^” Significance at *p* < 0.05 between genotypes
**TRITERPENOIDS**								
(Dihydroxy-urs-12-3n-28-oic acid)	471.3474	471.3515	−8.8	76.9	_Triterpenoid_471_2	444964656	386003980	
(Trihydroxy-urs-12-en-28-oic acid)	487.3424	487.3465	−8.5	64.9	_Esculentic acid	183735512	300310905	
(Dihydroxy-urs-12-3n-28-oic acid)	471.3474	471.3520	−9.8	76.5	_Triterpenoid_471_1	239898362	257066843	
(Trihydroxy-urs-12-en-28-oic acid)	487.3423	487.3410	2.6	66.7	_Triterpenoid_487_2	43279565	71329308	
(3-oxo-1?-hydroxy-urs-12-en-28-oic acid)	469.3318	469.3317	0.2	75.1	_Pomonic acid	50216895	36395157	
Ursolic acid	455.3524	455.3559	−7.7	79.5	Ursolic acid	44839049	25577316	
(3-oxo-19α-dihydroxy-urs-12-en-28-oic acid)	485.3267	485.3298	−6.3	66.9	_Triterpenoid_485	7363407	11435877	
(Oleaonolic acid)	455.3524	455.3559	−7.7	75.2	_Oleonolic acid	3884911	2638595	
(3-oxo-19α-dihydroxy-urs-12-en-28-oic acid)	485.3266	485.3266	0.1	69.6	_Annurcoic acid	2791337	1458985	
**PHLORIDZIN/PHLORIDZIN COMPONENTS**								
Phloridzin	435.1291	435.1325	−7.8	27.9	Phloridzin	3597822	29849517	^*^
Phloretin	273.0761	273.0704	20.9	42.2	Phloretin	8733671	21845333	
Phloretic acid	165.0551	165.0567	−9.9	10.1	Phloretic acid	343892	716204	
Phloroglucinol	125.0238	125.0264	−20.6	2.6	Phloroglucinol	308726	352251	
**FLAVONOIDS/FLAVONOID GLYCOSIDES**								
Epicatechin	289.0711	289.0729	−6.0	10.2	Epicatechin	27960574	20283468	
Hyperin	463.0880	463.0890	−2.1	21.8	Hyperin	6852133	3916945	
Quercitrin	447.0931	447.0937	−1.3	26.2	Quercitrin	2455612	933915	
Isoquercitrin	463.0871	463.0891	−4.4	21.8	Isoquercitrin	294231	649031	
Kaempferol-2-rutinoside	593.1502	593.1535	−5.6	25.8	Kaempferol2rut	182784	442723	
kaempferol-3-glucoside	447.0930	447.0934	−0.9	29.7	Kaempferol3gluc	17434	369714	^*^
Catechin	289.0711	289.0725	−4.8	5.9	Catechin	206357	303534	
(Reynoutrin)	434.0850	434.0933	−19.2	50.5	_Reynoutrin	0	297345	
Rutin	609.1451	609.1472	−3.5	21.8	Rutin	57712	32809	
**CHLOROGENIC ACIDS OR COMPONENTS**								
Chlorogenic acid (3-O-caffeoylquinic acid)	353.0872	353.0895	−6.6	6.5	Chlorogenic acid	19523743	15623595	
Quinic acid	191.0551	191.0572	−11.2	1.6	Quinic acid	20978217	10633187	
(P-coumaroylquinic acid)	337.0921	337.0943	−6.4	10.3	_Pcouquin acid	18129527	7244580	
(P-coumaroylquinic acid)	337.0921	337.0941	−5.9	8.0	_Pcouquin_unk1	3116356	5048331	
Caffeic acid	179.0341	179.0373	−17.8	7.3	Caffeic acid	1793336	1551952	
(P-coumaroylquinic acid)	337.0921	337.0942	−6.2	13.3	_Pcouquin_unk2	6181379	1154323	^*^
4-p-coumaric acid	163.0394	163.0411	−10.3	13.9	P-coumaric acid	958879	583182	
**BENZOIC ACID AND DERIVATIVES**								
(4-hydroxbenzoic acid) fragment	137.0238	137.0260	−16.1	15.6	4HBunk1	1789184	804328	^*^
(P-hydroxybenzoic acid alkyl ester)	285.0611	285.0557	19.0	5.1	_Uralenneoside	1628920	1143132	
3,4 hydroxybenzoic acid	153.0191	153.0220	−19.0	2.8	34HB	2225856	1639588	
4-hyrdroxybenzoic acid (authentic standard)	137.0238	137.0291	−38.6	3.2	4HB	7519629	2557597	
Benzoic acid	121.0289	121.0310	−17.5	14.5	Benzoic acid	825223	3678816	
**MISC PHENOLICS**								
(Phenolic glycoside)	Unknown	461.1672		7.2	_Dicaffeoylacteoside	2126495	1275547	
Gallic acid	169.0141	169.0166	−14.9	1.7	Gallic acid	493283	910044	
Hydrocaffeic acid	181.0501	181.0531	−16.7	6.8	Hydrocaffeic acid	700079	602926	
Salicin	285.0971	285.1004	−11.6	4.8	Salicin	122619	182760	
**COUMARINS**								
(Esculin)	339.0712	339.0747	−10.3	5.2	_Esculin	213443	660061	^*^
Scopoletin	191.0350	191.0371	−11.0	15.2	Scopoletin	56206	61686	
**BENZOFURANS/DIBENZOFURANS**								
(Eriobofuran)	243.0740	243.0663	31.7	53.1	_Eriobo2	2365538	2857828	
(Noraucuparin)	215.0712	215.0711	0.5	27.3	_Noraucuparin	388993	883580	
(Aucuparin)	229.0861	229.0861	0.2	2.1	_Aucuparin	130917	72510	
(Eriobofuran)	243.0661	243.0671	−4.1	41.3	_Eriobo1	74616	656321	^*^
**PROCYANIDINS**								
Procyanidin B2	577.1345	577.1367	−3.8	8.0	Procyanidin B2	511461	774127	
(Procyanidin)	577.1345	577.1367	−3.8	6.4	_Procyanidin_6_55	2743098	758511	
(Procyanidin)	865.1981	865.2016	−4.0	12.3	_ProcyC1	169764	142725	
(Procyanidin)	577.1345	577.1370	−4.3	21.4	_Procyanidin_21_48	0	58335	

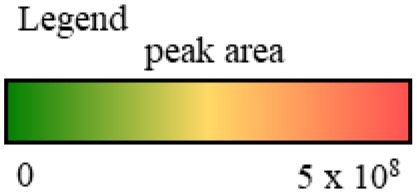

Color coding in peak area cells indicates relative concentration. Significance at p < 0.05 between genotypes (^*^).

Tentative identification of triterpenoids was based on agreement of accurate mass with ursenoic acids in apple peel reported by McGhie et al. (2012) as well as elemental composition per accurate mass and expected isotope ratios generated in Agilent MassHunter software. Furthermore, an authentic standard of ursolic acid confirmed this compound, and MS/MS data generated by 10, 20, 40 V collision energies supported similar mass spectra for other triterpenoids. The present analysis did not identify the positions of additional hydroxyl or oxo groups nor their configuration, and compounds are presented as types of ursenoic acids accordingly. In the present study, there were masses in agreement with 5 of the 8 major triterpenoid ion traces presented by McGhie et al. (2012). Since samples were analyzed in negative ionization mode via ESI, which has relatively softer ionization (Wolfender, 2009), the tentative triterpenoids presented as the deprotonated molecular ion (pseudomolecular) ion (M-H)^−^. The ion traces present in this study corresponding to major traces by McGhie et al. (2012) includes monohydroxy ursenoic acids (*m/z* 455.3524, [C_30_H_48_O_3_–H]^−^), dihydroxyursenoic acids (*m/z* 471.3474, [C_30_H_48_O_4_–H]^−^), oxohydroxy ursenoic acids (*m/z* 469.3318; [C_30_H_46_O_4_–H]^−^), trihydroxy ursenoic acids (*m/z* 487.3423; [C_30_H_48_O_5_–H]^−^), and oxodihydroxy ursenoic acids (*m/z* 487.3418; [C_30_H_46_O_5_–H]^−^).

### Validation of rhizodeposit production in connection with plant growth

Summed peak areas from analysis of polar compounds (zicpHILIC) and phenolic compounds indicated that trees treated with the toxic compound butyrolactone had reduced production of metabolites (Figure 2). *De novo* compound lists from the untargeted MZmine workflow were analyzed in MetaboAnalyst (Supplemental Figure 1). Statistically differing metabolites (identified and unidentified) are indicated in Supplemental Table 4: rhizodeposit production validation.xlsx).

### Endophyte assessment

Fungi were detected by qPCR in 30% of the samples (Supplemental Table 5: qPCR results for micropropagated tree roots endophyte assessment.xlsx). Analysis of results based on universal fungal primers indicated the presence of fungal amplicons the same length as internal positive controls in approximately 30% of the samples with cycle threshold values exceeding background noise. For bacterial primers, non-specific amplification of plastid DNA is expected to have contributed to results. One segment (18 segments per plate, one plate per tree) from one tree was noted to have fungal growth but no fruiting structures were observed to facilitate identification. No bacterial growth was observed in response to streaking root segments across TSA plates.

## Discussion

Previous work indicated that apple rootstock genotype impacts rhizodesposit quantity and composition, and is also influenced by environmental, physiological, and ontological factors (Leisso et al., 2017). Other research indicates that single rhizodeposit compounds can alter soil microbial community composition resulting in changes to metabolite degradation (Lu et al., 2017), which could have implications for the role of some apple root compounds involved in alleopathy to growth of apple seedlings. The present study expands the list of apple rhizodeposit phenolic compounds previously reported (Leisso et al., 2017) and further indicates there are a number of sugars, organic acids, amino acids, sugar alcohols, putative triterpenoids, and potentially additional classes of phenolic compounds present in apple rhizodeposits. This work provides the basis for continued assessment of apple rhizodeposits on soil microbial communities by indicating some of the major compounds and their relative levels present in apple rhizodeposits in an assessment conducted in the absence of microbial activity.

In the current study, several organic acids were detected at relatively high concentration, including malic acid, succinic acid, malonic acid, and oxalic acid. Functionally, organic acids released by roots into the rhizosphere can influence nutrient availability through changes in soil pH. For example, salicylic and citramalic acid are found in high concentrations in sugar beet rhizodeposits and are able to solubilize phosphorus (Khorassani et al., 2011). Soil microbes can also produce organic acids and alter substrate pH (Hoberg et al., 2005; Mohammadi, 2012) and composition of the rhizosphere microbial community may differ according to rootstock genotype (Mazzola and Manici, 2012; Reed and Mazzola, 2015). Malic acid, the major organic acid present in apple fruit tissue, has been reported to function in the recruitment of potentially plant-beneficial bacteria to the rhizosphere (Rudrappa et al., 2008). However, this metabolite was not detected in rhizodeposits at exceptionally high relative levels in the present study. Apple rootstock genotype affects mineral nutrient concentration of above ground foliage (Fazio et al., 2015) and similar genotype effects exist in other crops (Rengel and Marschner, 2005), but relative influence of genotype specific-root exudation versus rhizosphere soil microbial community activity on foliar nutrient concentrations remains unknown. An especially intriguing aspect of organic acid rhizodeposits is that the impact of these compounds on nutrient availability depends on both the specific organic acid as well as the soil type where these interactions take place (Wang et al., 2016).

Urea levels were unexpectedly high in rhizodeposits but did not differ significantly between genotypes; this metabolite was not assessed in the previous study (Leisso et al., 2017). In plant tissue, this metabolite results from either root uptake of urea or catabolism of arginine (Witte, 2011). The media in which the micropropagated trees were grown contained ammonium nitrate, which could ultimately be the source of the urea in the micropropagated trees. However, levels of this metabolite were not consequential in the REM or neoprene float quality control assessments. The mechanism of urea release in the present study is unclear.

Among identified phenolic compounds, phloridzin, kaemferol-3-glucoside, and esculin differed in relative quantity between G935 and M26. Phloridzin has been reported repeatedly as a major phenolic component of apple roots and bark and yet remains ambiguous in terms of impact on pathogen and other microbial populations. Some studies have reported phloridzin to possess detrimental allelopathic effects on apple seedlings (Yin et al., 2018), while others indicate levels increase in plants in response to challenge by root pathogens (Hoffman et al., 2009). It has also been reported to be utilized as a carbon source for a foliar apple pathogen (*Venturia inequalis*) (Hunter, 1975). Similar to our previous report (Leisso et al., 2017), in the present study phloridzin levels were higher in M26 rootstock which generally supports higher populations of root infesting pathogens (Mazzola et al., 2009) and has demonstrated greater susceptibility to apple replant disease in field trials relative to G935 (Fazio et al., 2006). Microbial degradation products of phloridzin (including phloroglucinol, phloretin, and phloretic acid) (Chatterjee and Gibbins, 1969; Jayasankar et al., 1969) were also assessed in this study but did not differ significantly between genotypes. Phloretin has inhibitory activity against apple pathogens *in vitro* (Shim et al., 2010).

Chlorogenic acid, quinic acid, and epicatechin were additional phenolic compounds found in relatively high levels in rootstock rhizodpeposits. Epicactechin has been reported in high levels in other rosaceous plants (Oszmianski et al., 2007) and found to inhibit infection of coffee by the foliar fungal pathogen *Colletotrichum kahawae* (Chen et al., 2006). Many of the phenolic compounds assessed in the present study have either pathogen inhibition (Lanoue et al., 2010), antioxidant properties (Hamauzu et al., 2005) (chlorogenic acid), or anitproliferative and pro-apoptotic effects (Mari et al., 2010) (chlorogenic acid, procyanidins, flavonols, dihydrochalcones). The impact of many of these compounds on soil microorganisms and activity of apple root pathogens remains to be assessed.

An intriguing result is the relatively high levels of putative triterprenoids present in micropropagated tree rhizodeposits. The structure of the compounds detected in the present study were not fully elucidated but the masses correspond to triterpenoid compounds reported in apple peel tissue (McGhie et al., 2012). In the present study, only ursolic acid could be confirmed, due to the inavailability of ursane triterpenoid standards. Triterprenoids can have roles as plant defense compounds, including inhibition of microbial growth and insect herbivory, and can be involved in interspecies signaling (Sindambiwe et al., 1998; Gershenzon and Dudareva, 2007; Sawai and Saito, 2011; Moghaddam et al., 2012). Reports of triterpenoid exudation from roots are not extensive, although a smaller tricylic triterpene has been reported to be exuded from Arabidopsis roots and implicated in defense against *Pythium irregulare* (Sohrabi et al., 2017). Additional compounds, which may function as phytoalexins, classified as benzofurans/dibenzofurans based on mass spectral features and previously reported from apple tissues (Chizzali et al., 2012), were detected, but identity was not confirmed due to the inability to obtain authentic standards.

The question remains as to which compounds or classes of compounds has the most influence on the rhizosphere microbiome in terms of functions that impact tree health and apple fruit production. In *Arabidopsis*, the application of phenolic compounds resulted in a greater number of unique OTUs compared with other groups of compounds, including sugars, sugar alcohols, and amino acids (Badri et al., 2013). However, the functional consequences for plant health and growth were not examined. Furthermore, specific compounds (salicylic acid and GABA) had broad impacts on populations in several microbial groups (Badri et al., 2013), a concept that we are currently testing with phloridzin. Yet in the present study, a greater number of sugars and organic acids differed between the genotypes (G935 and M26) than phenolic compounds. However, the present study was relatively small in scale and included trees and tree roots at a very young age with assessment of rhizodeposit composition conducted at a single timepoint. Previous work with older and larger commercial trees indicates that rhizodeposit compositional changes occur in response to both time and environment (Leisso et al., 2017). Potential implications of findings from the present study should be extended with consideration of these factors. We hypothesize changes in rhizodeposit composition as roots and leaves age, and in response a more demanding environment outside axenic conditions under which the current study was conducted. The utility of results presented here is that rhizodeposits were assessed from axenically micropropagated trees where the influence of microbial metabolites (which can be considerable; Leisso et al., 2017) was (with the possible exception of endophytic contribution) avoided.

Another point in consideration of rhizodeposit assessment is the biochemical/physical relationship roots have with their external media in terms of rhizodeposit release. The large solute concentration gradient in the root, relative to the external medium, likely causes molecules with no charge, like sugars, to be lost through passive diffusion (Jones et al., 2004). This study was performed in sterile hydroponic (water-based) conditions, where the solute concentration outside the root is initially very low and would imply rapid diffusion of some compounds from the root into the water. Counter intuitively, in hydroponic set-ups where metabolites are not lost due to microbial degradation, previous studies indicate that when there is no microbial degradation, some metabolites are actively taken back up by the plant, thus suggesting that sterile hydroponic methods actually underestimate the total quantities of rhizodeposits (Jones et al., 2004).

Endophyte analyses were inconclusive, which was somewhat unexpected, as trees had been propagated from actively growing shoot tips of trees, and because until recently (Liu et al., 2018), endophytes had been reported only from apple roots (Bulgari et al., 2012). We cannot exclude the possible influence of tree endophytes on the results presented in this report. However, trees originated from the same propagation lines, so it could be expected that any endophytic communities would be similar and have similar influences on rhizodeposits. Whether or not any microbial endophytes present in apple trees have consequential influence on rhizodeposits remains to be determined, although endophyte presence has been reported to impact root exudate profiles in other plant species (Guo et al., 2015).

Our findings confirmed that vegetative activity contributes to the levels of bulk rhizodeposits, indicating both that rhizodeposits may be translocated from photosynethic activity in the leaves and that sloughed off root tissue or cells are not likely to be the only source of rhizodeposits. This concurs with our previous results where total abundance of rhizodeposit metabolites was well correlated with total leaf area (Leisso et al., 2017).

## Conclusion

This study reports methods for axenic collection of apple rhizodeposits, and describes the relative levels of a number of compounds in collected rhizodeposits from two apple rootstock genotypes (G935 and M.26), including phenolic compounds, sugars, sugar alcohols, amino acids, organic acids, and several putative triterpenoids. Potential impacts of these metabolites in terms of plant health include their function as substrate sources for beneficial microorganisms or in demonstrating inhibitory activity toward various plant pathogens. A prospective long-term vision for this line of research is to integrate apple genotype-specific rhizodeposition patterns or capacity with particular soil characteristics to enable site-suitability decisions for apple rootstock genotypes or appropriate site modifications to enhance horticultural output. To achieve this, additional research is needed to (1) further delineate rhizodeposit metabolites that are consistently produced at levels high enough to have functional impacts on both soil chemistry and microbial ecology, (2) determine the effects of major rhizodeposits on microbial communities, including identifying endophytes and their contributions to rhizodeposits, (3) further determine the primary environmental, ontological, and physiological drivers that change levels of rhizodeposits, and (4) integrate rootstock genotype, rhizodeposition, rhizosphere microbiome, and soil chemistry to enable precision agricultural determination of appropriate system modifications.

## Author contributions

RL designed and carried out the research. DR contributed methods for instrumental analysis and MM contributed to experimental design and writing.

### Conflict of interest statement

The authors declare that the research was conducted in the absence of any commercial or financial relationships that could be construed as a potential conflict of interest.
