# Supplementary figures and images for: Targeted Metabolic Profiling Indicates Apple Rootstock Genotype-Specific Differences in Primary and Secondary Metabolite Production and Validate Quantitative Contribution From Vegetative Growth
Source: Front Plant Sci. 2018 Sep 21;9:1336. doi: 10.3389/fpls.2018.01336 (PMC6160592; doi:10.3389/fpls.2018.01336)

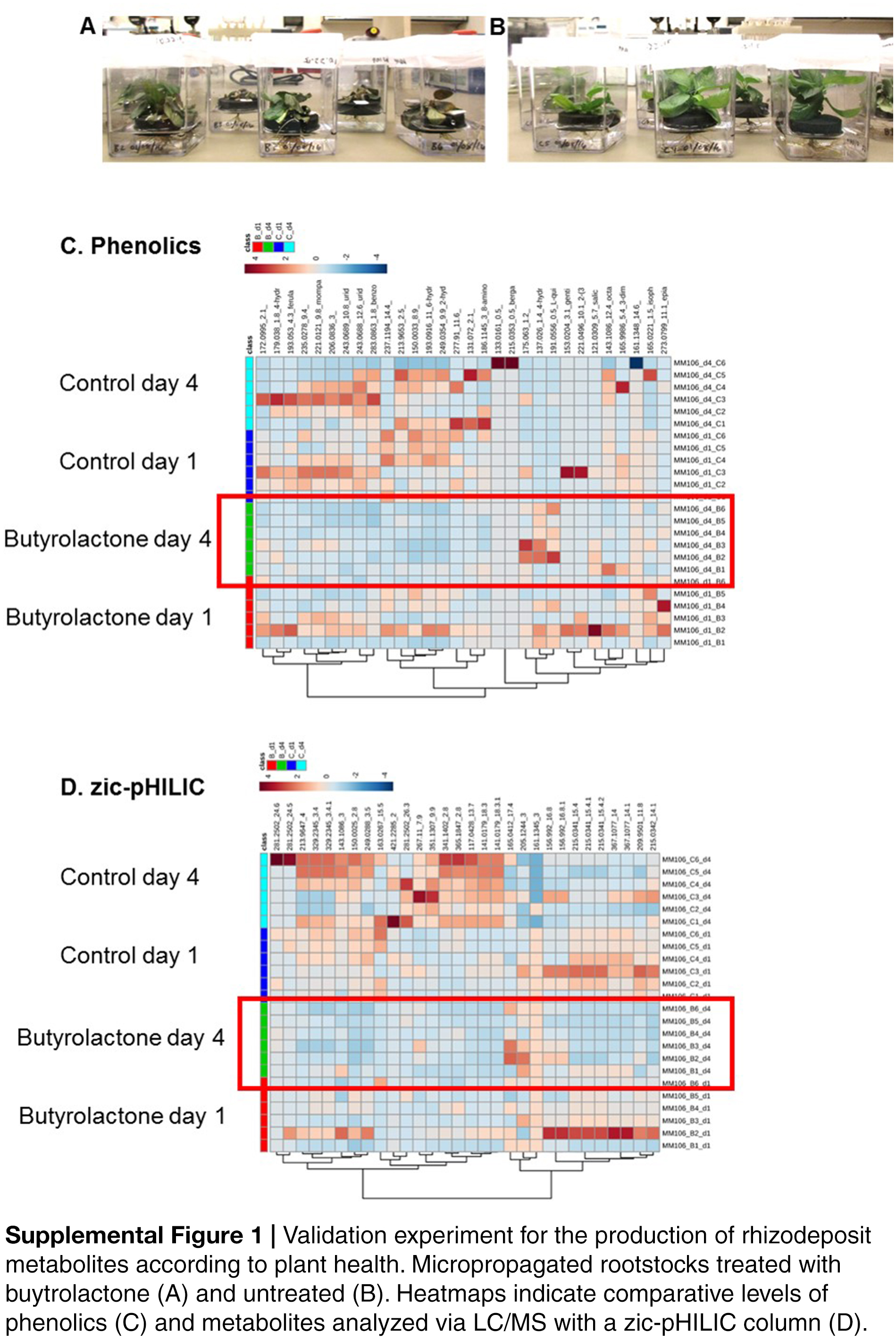

Supplement: Supplementary file 6 [file Image_1.JPEG]
